# The complete chloroplast genome of critically endangered *Chimonobambusa hirtinoda* (Poaceae: *Chimonobambusa*) and phylogenetic analysis

**DOI:** 10.1038/s41598-022-13204-2

**Published:** 2022-06-10

**Authors:** Yanjiang Liu, Xiao Zhu, Mingli Wu, Xue Xu, Zhaoxia Dai, Guangqian Gou

**Affiliations:** 1 Key Laboratory of Plant Resource Conservation and Germplasm Innovation in Mountainous Region (Ministry of Education), College of Life Sciences/Institute of Agro-Bioengineering, Guizhou University, Guiyang, 550025 Guizhou Province China; 2 Bamboo Research Institute, Guizhou University, Guiyang, 550025 China; 3 College of Forestry, Guizhou University, Guiyang, 550025 China

**Keywords:** Developmental biology, Plant sciences, Systems biology, Ecology

## Abstract

*Chimonobambusa hirtinoda,* a threatened species, is only naturally distributed in Doupeng Mountain, Duyun, Guizhou, China. Next-generation sequencing (NGS) is used to obtain the complete chloroplast (cp) genome sequence of *C. hirtinoda*. The sequence was assembled and analyzed for phylogenetic and evolutionary studies. Additionally, we compared the cp genome of *C. hirtinoda* with previously published *Chimonobambusa* species. The cp genome of *C. hirtinoda* has a total length of 139, 561 bp and 38.90% GC content. This genome included a large single -copy (LSC) region of 83, 166 bp, a small single-copy (SSC) region of 20, 811 bp and a pair of inverted repeats of 21,792 bp each. We discovered 130 genes in the cp genome, including 85 protein-coding genes, 37 tRNA, and 8 rRNA genes. A total of 48 simple sequence repeats (SSRs) were detected. The A/U preference of the third nucleotide in the cp genome of *C. hirtinoda* was obtained by measuring the codon usage frequency of amino acids. Furthermore, phylogenetic analysis using complete cp sequences and *matK* gene revealed a genetic relationship within the *Chimonobambusa* genus. This study reported the chloroplast genome of the *C. hirtinoda*.

*Chimonobambusa* genus contains 37 species, 34 species distributed in China. Most of its bamboo shoots proliferate in autumn, which is delicious, nutrients and contain protein, amino acids and minerals^1,2^. Bamboo shoots are healthy and beneficial food for local people. Additionally, the bamboo is rich in cellulose pulp and is a high-quality raw material for papermaking, bamboo handicrafts, bamboo plywood, and craft furniture. As a result, *Chimonobambusa* has high economic, edible, and cultural value^3–5^.

*Chimonobambusa hirtinoda* was listed in the red catalogue by IUCN in 2007 and rated as a nationally endangered plant. *C. hirtinoda* is only naturally distributed in Doupeng Mountain, Duyun, Guizhou, China^6,7^. However, recently, with the tourism development in Doupeng Mountain and natural environmental losses, the habitat of *C. hirtinoda* has been damaged, and they are on the verge of extinction^8^. Thus, the natural resources of *C. hirtinoda* must be protected in all aspects. Most of the woody bamboos are primarily reproduced through rhizomes, with a random flowering period, and some species only bloom once in a lifetime^9^. The division of the Bambusoideae family is controversial because their classification and identification depend upon the morphological characteristics of vegetative organs. Consequently, their research from a morphological to molecular perspective is required to assess the classification and evolutionary relationship of bamboo species.

The origin of chloroplast (cp) is hypothesized to be obtained from cyanobacteria through endosymbiosis^10^. As the photosynthetic organelle of plant cells, chloroplast plays a key role in photosynthesis and has crucial implications for plant physiology and development^11,12^. The structure of the chloroplast genome is conservative, comprising a large single-copy (LSC) region, a small single-copy (SSC) region, and two inverted repeats (IRs) regions in opposite directions^13^. Therefore, chloroplast genomes have been widely used as DNA barcodes to identify species. Phylogenetic studies and chloroplast haplotypes are used to analyze the genetic diversity of species^14–16^.

Several reports are present on the chloroplast genome of the Arundinariatae in the Bambusoideae^17^, but limited data is available on the *Chimonobambusa* genus. Thus, this study reported the chloroplast genome of *C. hirtinoda*, including its gene content, codon usage, and its comparison with closed species. A phylogenetic relationship was constructed based on previously published cp genomes of Bambusoideae to clarify the taxonomic position of *C. hirtinoda*. These findings will provide valuable genetic resources for further research on the phylogenetic position of *C. hirtinoda* and investigate evolutionary relationships of the order Bambusoideae.

## Results

### Assembly and annotation of the chloroplast genomes

**Figure 1:**
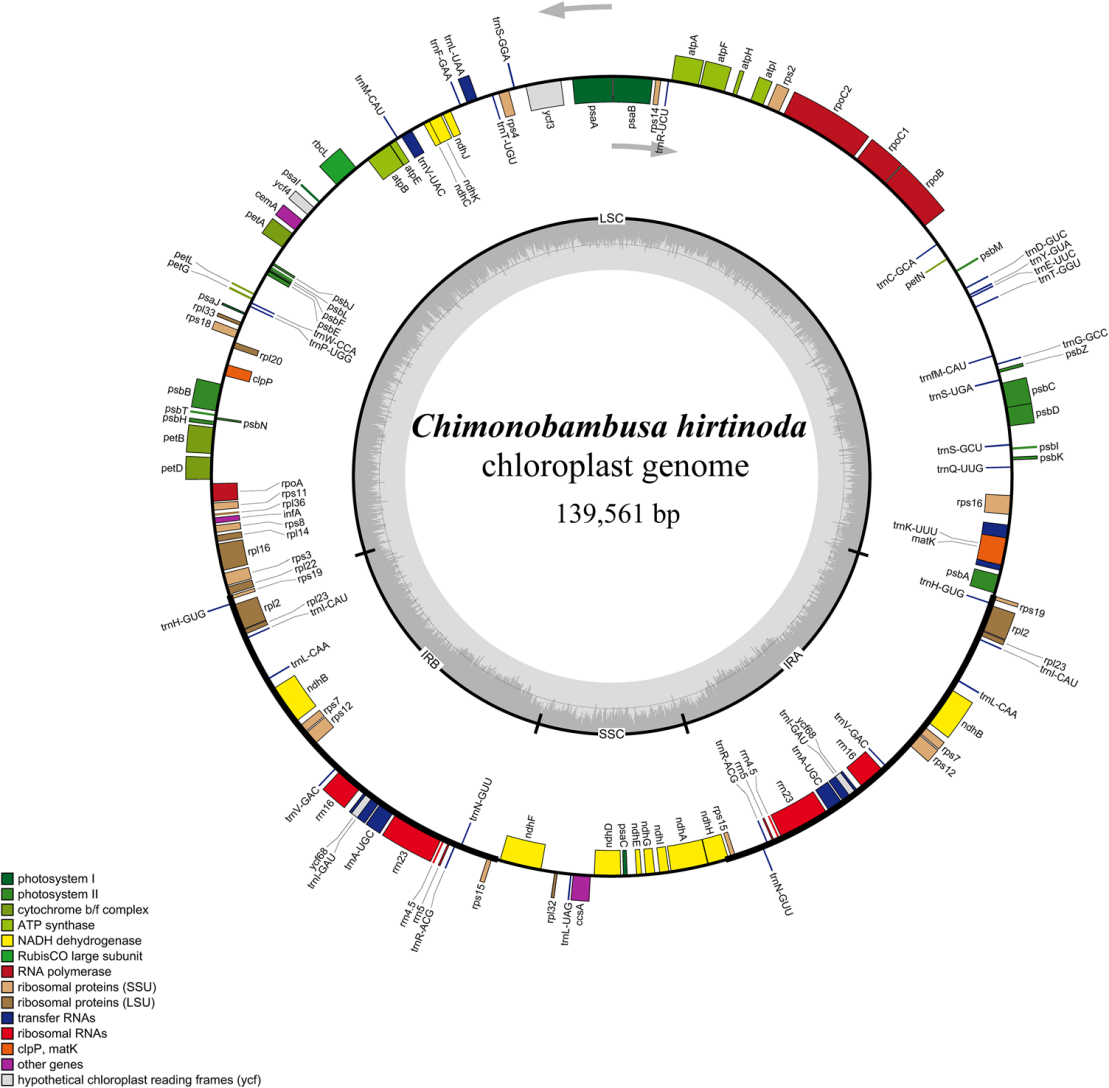
Chloroplast genome map of *C. hirtinoda*. Different colors represent different functional genes groups. Genes outside the circle indicate counterclockwise transcription, and genes inside the clockwise transcription. The thick black line on the outer circle represents the two IR regions. The GC content is the dark gray area within the ring.

Assembly resulted in a whole cp genome sequence of *C. hirtinoda* with a length of 139, 561 bp (Fig. 1), consisting of 83, 166 bp large single-copy region, 20, 811 bp small single-copy regions, and two 21,792 bp IR regions, comprising the typical quadripartite structure of terrestrial plants. The cp genome of *C. hirtinoda* was annotated with 130 genes, including 85 protein-coding genes, 37 tRNA genes, and 8 rRNA genes (Table 1). Most of the 15 genes in the *C. hirtinoda* cp genome contain introns. Of these, 13 genes contain one intron (*atpF*, *ndhA*, *ndhB*, *petB*, *petD*, *rpl2*, *rpl16*, *rps16*, *trnA-UGC*, *trnI-GAU*, *trnK-UUU*, *trnL-UAA*, *trnV-UAC*) and only the gene *cyf3* includes two introns, and the gene *clpP* intron was deleted (Supplementary Table S1). The *rps12* gene contained two copies, and the three exons were spliced into a trans-splicing gene^18^.

**Table 1:** Summary of the chloroplast genome of *C. hirtinoda*.

Genome features	*C. hirtinoda*
Genome size (bp)	139,561
LSC size (bp)	83,166
SSC size (bp)	12,811
IR size (bp)	21,792
GC content (%)	38.9%
No. of genes	130
No. of PCGs	85
No. of tRNA	37
No. of rRNA	8

The *accD*, *ycf1,* and *ycf2* genes were missing in the cp genome of *C. hirtinoda*, and the introns in the genes *clpP* and *rpoC1* were lost. This phenomenon is consistent with previous systematic evolutionary studies on the genome structure of plants in the Poaceae family^19^. The phenomenon of missing genes is reported in other plants^20–23^.

The total GC content in the *C. hirtinoda* cp genome was 38.90%, and the content for each of the four bases, A, T, G, and C, was 30.63%, 30.46%, 19.57%, and 19.33%, respectively (Table 2). The LSC region (36.98%) and SSC region (33.21%) exhibited much lower values than the IR region (44.23%), indicating a non-uniform distribution of the base contents in the cp genome, probably because of four rRNAs in the IR region, which in turn makes the GC content higher in the IR region. These values were similar to cp genome results previously reported for some Poaceae plants^24,25^.

**Table 2:** Base composition in the *C. hirtinoda* choloroplast genome.

Region	Length (bp)	A (%)	T (%)	G (%)	C (%)	GC (%)
LSC	83,166	31.24	31.78	18.76	18.22	36.98
SSC	12,811	36.02	30.78	16.17	17.04	33.21
IRA	21,792	27.96	27.81	21.19	23.04	44.23
IRB	21,792	27.96	27.81	21.19	23.04	44.23
Total genome	139,561	30.63	30.46	19.57	19.33	38.90
CDS	60,531	29.63	30.85	21.20	18.3	39.53

### Repeat sequences and codon analysis

SSR consists of 10-bp-long base repeats and is widely used for exploring phylogenetic evolution and genetic diversity analysis^26–29^.

**Figure 2:**
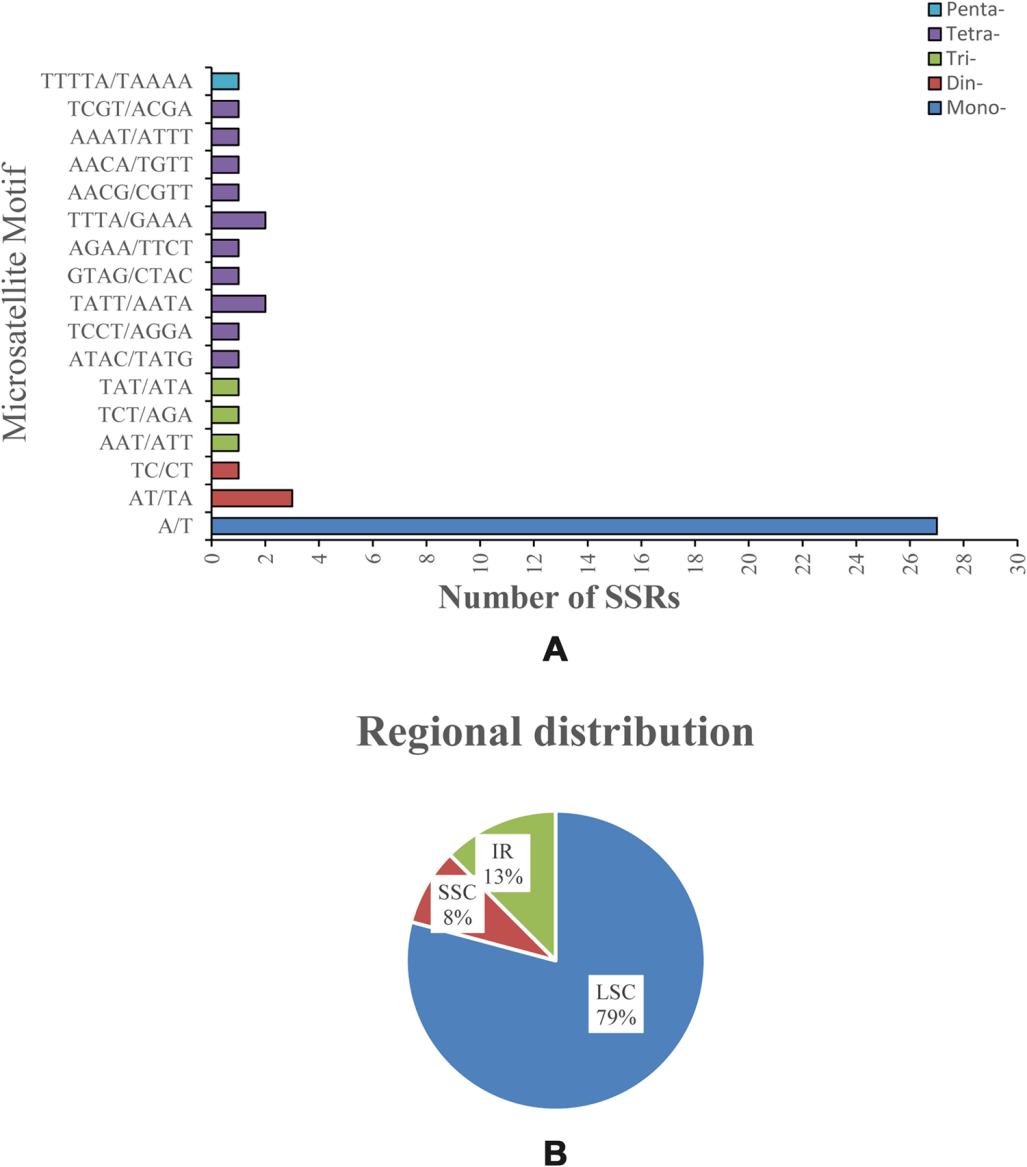
Analysis of simple sequence repeats in *C. hirtinoda* cp genome. (**A**) The percentage distribution of 45 SSRs in LSC, SSC, and IR regions. (**B**).

In total, 48 SSRs were detected in *C. hirtinoda*, including 27 mononucleotide versions, accounting for 56.25% of the total SSRs, primarily consisting of A or T. Additionally, four dinucleotide repeats consisting of AT/TA and TC/CT repeats, and 3 tri, 13 tetra, and 1penta-repeats (Fig. 2A). From the SSRs distribution perspective, the majority (79%) of SSRs (38) were observed in the LSC area, whereas 6 SSRs in the IR region (13%) and 4 SSRs in the SSC region (8%) were discovered (Fig. 2B). Previous research suggests that the distribution of SSRs numbers in each region and the differences among locations in GC content are related to the expansion or contraction of the IR boundary^30^.

**Figure 3:**
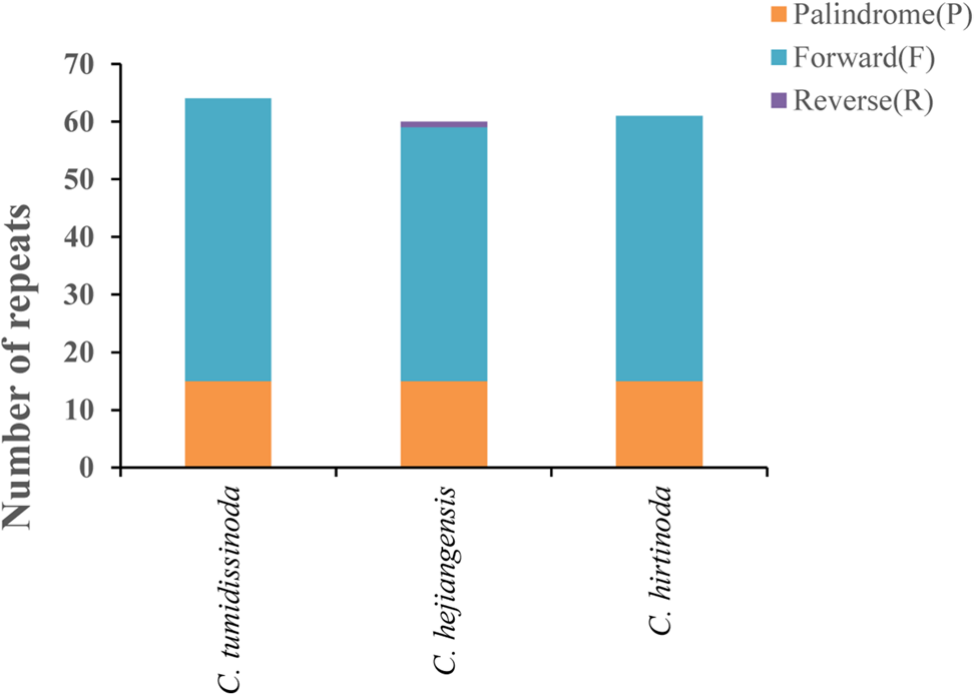
Information of chloroplast genome repeats of *Chimonobambusa* genus species.

The REPuter program revealed that the cp genome of *C. hirtinoda* was identified with 61 repeats, consisting of 15 palindromic, 19 forward and no reverse and complement repeats (Fig. 3). We noticed that repeat analyses of three *Chimonobambusa* genus species exhibited 61–65 repeats, with only one reverse in *C. hejiangensis.* Most of the repeat lengths were between 30 and 100 bp, and the repeat sequences were located in either IR or LSC region^31^ (Supplementary Table S2).

**Figure 4:**
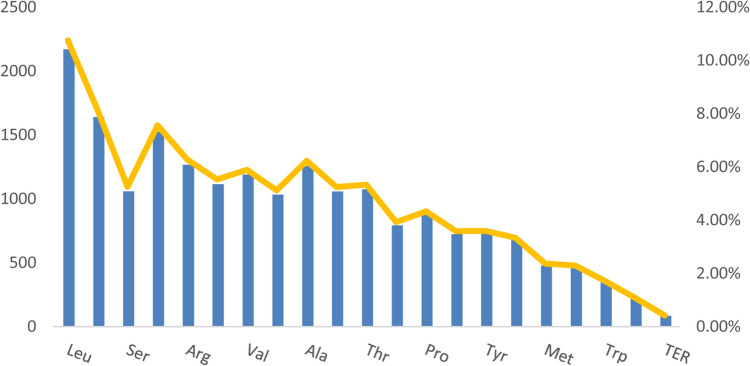
Amino acid frequencies in *C. hirtinoda* cp genome protein coding sequences. The column diagrams indicate the number of amino acid codes, and the broken line indicates the proportion of amino acid codes.

We identified 20,180 codons in the coding region of *C. hirtinoda* (Fig. 4, Supplementary Table S3). The codon AUU of Ile was the most used, and the TER of UAG was the least used codon (817 and 19), excluding the termination codons. Leu was the most encoded amino acid (2,170), and TER was the lowest (85). The Relative Synonymous Codon Usage (RSCU) value greater than 1.0 means a codon is used more frequently^32^. The RSCU values for 31 codons exceeded 1 in the *C. hirtinoda* cp genome, and of these, the third most frequent codon was A/U with 29 (93.55%), and the frequency of start codons AUG and UGG used demonstrated no bias (RSCU = 1).

### Comparative analysis of genome structure

**Figure 5:**
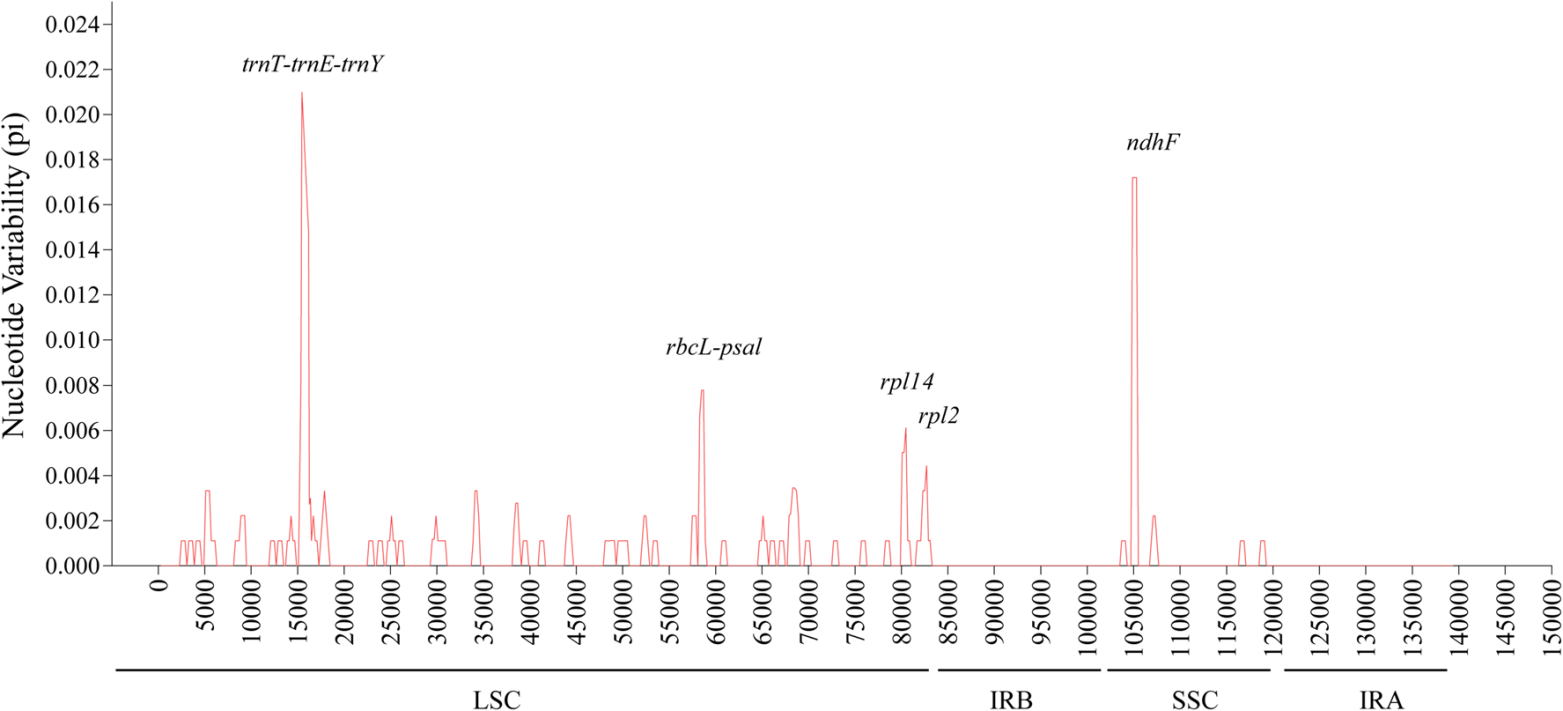
Sliding window analysis of *Chimonobambusa* genus complete chloroplast genome sequences. X-axis: position of the midpoint of a window, Y-axis: nucleotide diversity of each window.

The nucleotide variability (Pi) values of the three cp genomes discovered in the *Chimonobambusa* genus species ranged from 0 to 0.021 with an average value of 0.000544, as demonstrated from DnaSP 5.10 software analysis. Five peaks were observed in the two single-copy regions, and the highest peak was present in the *trnT-trnE-trnY* region of the LSC region (Fig. 5). The Pi value for LSC and SSC is significantly higher than that of the IR region. In the IR region, highly different sequences were not observed, a highly conserved region. The sequences of these highly variable regions are reported in other plants during examinations for species identification, phylogenetic analysis, and population genetics research^33–35^.

**Figure 6:**
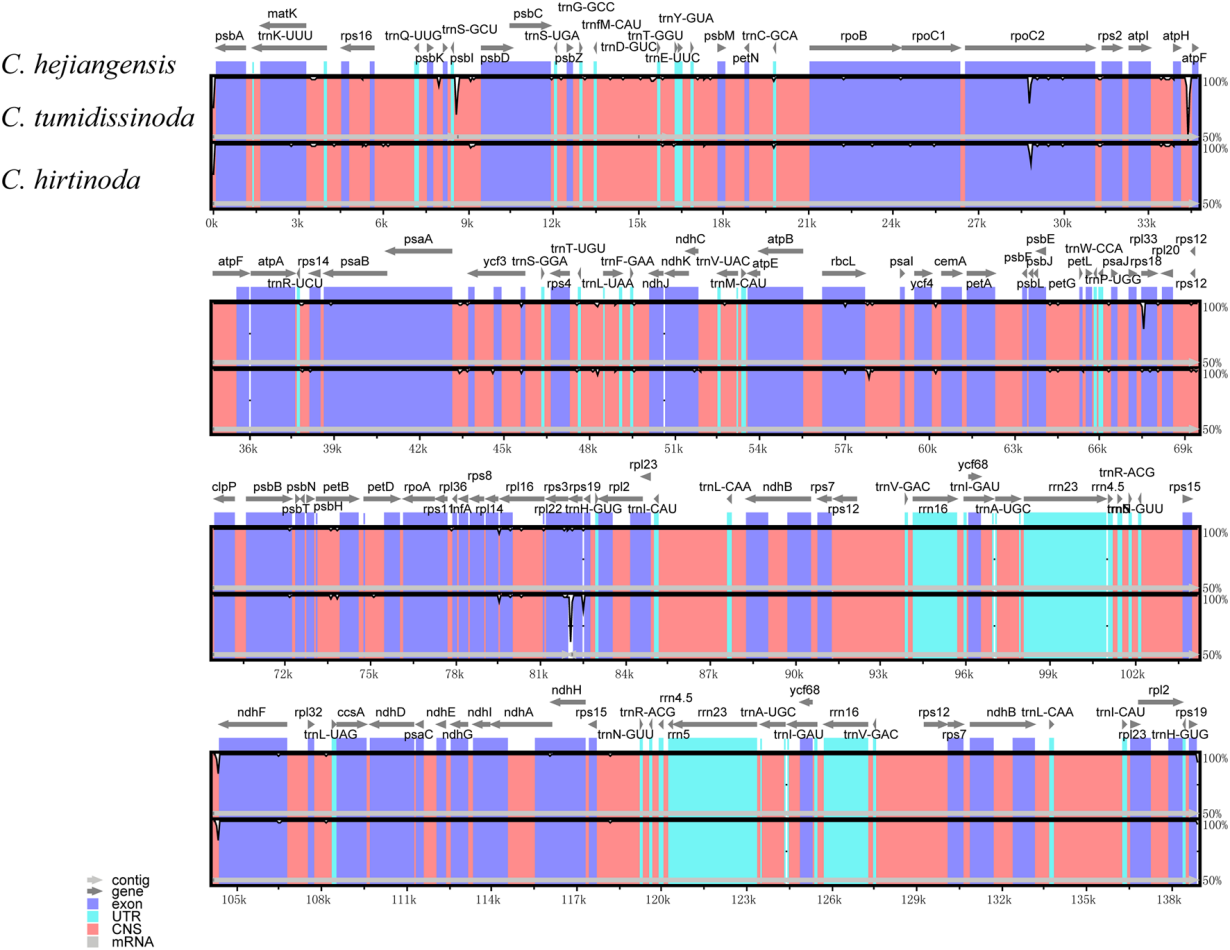
Visualization of genome alignment of three species chloroplast genome sequences using *Chimonobambusa hejiangensis* as reference. The vertical scale shows the percent of identity, ranging from 50 to 100%. The horizontal axis shows the coordinates within the cp genome. Those are some colors represents protein coding, intron, mRNA and conserved non-coding sequence, respectively.

The structural information for the complete cp genomes among three *Chimonobambusa* genus species revealed that the sequences in most regions were conserved (Fig. 6). The LSC and SSC regions exhibit a remarkable degree of variation, higher than the IR region, and the non-coding region demonstrates higher variability than the coding region. In the non-coding areas, 7–9 k, 28–30 k, 36 k and other gene loci differed significantly. Genes *rpoC2*, *rps19*, *ndhJ* and other regions differ in the protein-coding region. However, the agreement between the tRNA and rRNA regions is 100%. A similar phenomenon has also been reported by others^36^.

### IR contraction and expansion in the chloroplast genome

Due to the unique circular structure of the cp genome, there are four junctions between the LSC/IRB/SSC/IRA regions. During species evolution, the stability of the two IR regions sequences was ensured by the IR region of the chloroplast genome expanding and contracting to some degree, and this adjustment is the primary reason for chloroplast genome length variation^37,38^.

**Figure 7:**
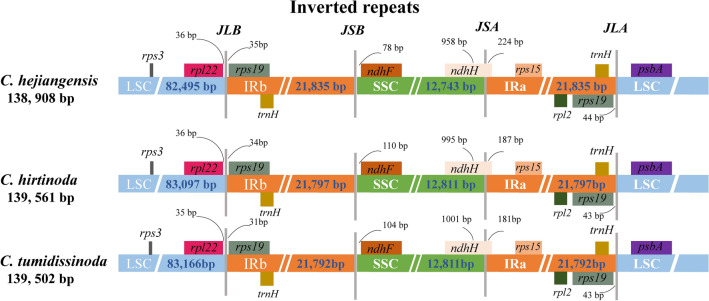
Comparison of LSC, SSC and IR boundaries of chloroplast genomes among the three *Chimonobambusa* species. The LSC, SSC and IRs regions are represented with different colors. JLB, JSB, JSA and JLA represent the connecting sites between the corresponding regions of the genome, respectively. Genes are showed by boxes.

The variations at IR/SC boundary regions in the three *Chimonobambusa* genus chloroplast genomes were highly similar in the organization, gene content, and gene order. The size of IR ranges from 21,797 bp (*C. tumidissinoda*) to 21,835 bp (*C. hejiangensis*). The *ndhH* gene spans the SSC/IRa boundary, and this gene extended 181–224 bp into the IRa region for all three *Chimonobambusa* genus. The gene *rps19* was extended from the IRb to the LSC region with a 31–35 bp gap. The *rpl12* gene was located in the LSC region of all genomes, varied from 35–36 bp apart from the LSC/IRb (Fig. 7).

**Figure 8:**
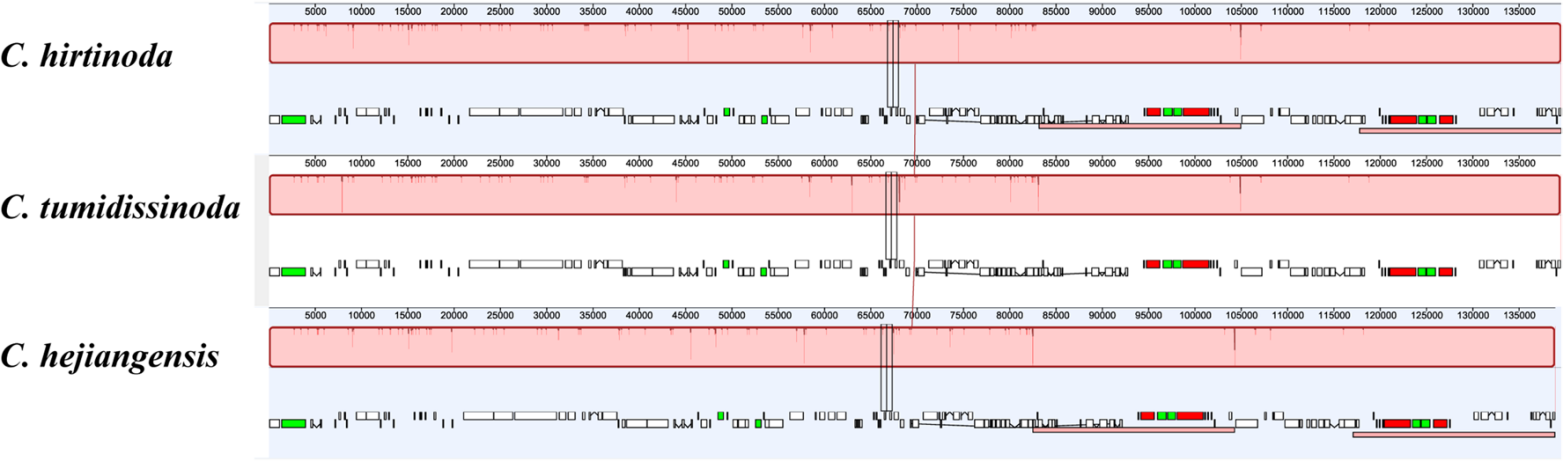
The chloroplast genomes of three *Chimonobambusa* species rearranged by the software MAUVE. Locally collinear blocks (LCBs) are represented by the same color blocks connected by lines. The vertical line indicates the degree of conservatism among position. The small red bar represents rRNA.

Three chloroplast genomes of the *Chimonobambusa* genus were compared using the Mauve alignment. The results showed that all sequences show perfect synteny conservation with no inversion or rearrangements (Fig. 8).

### Phylogenetic analysis

**Figure 9:**
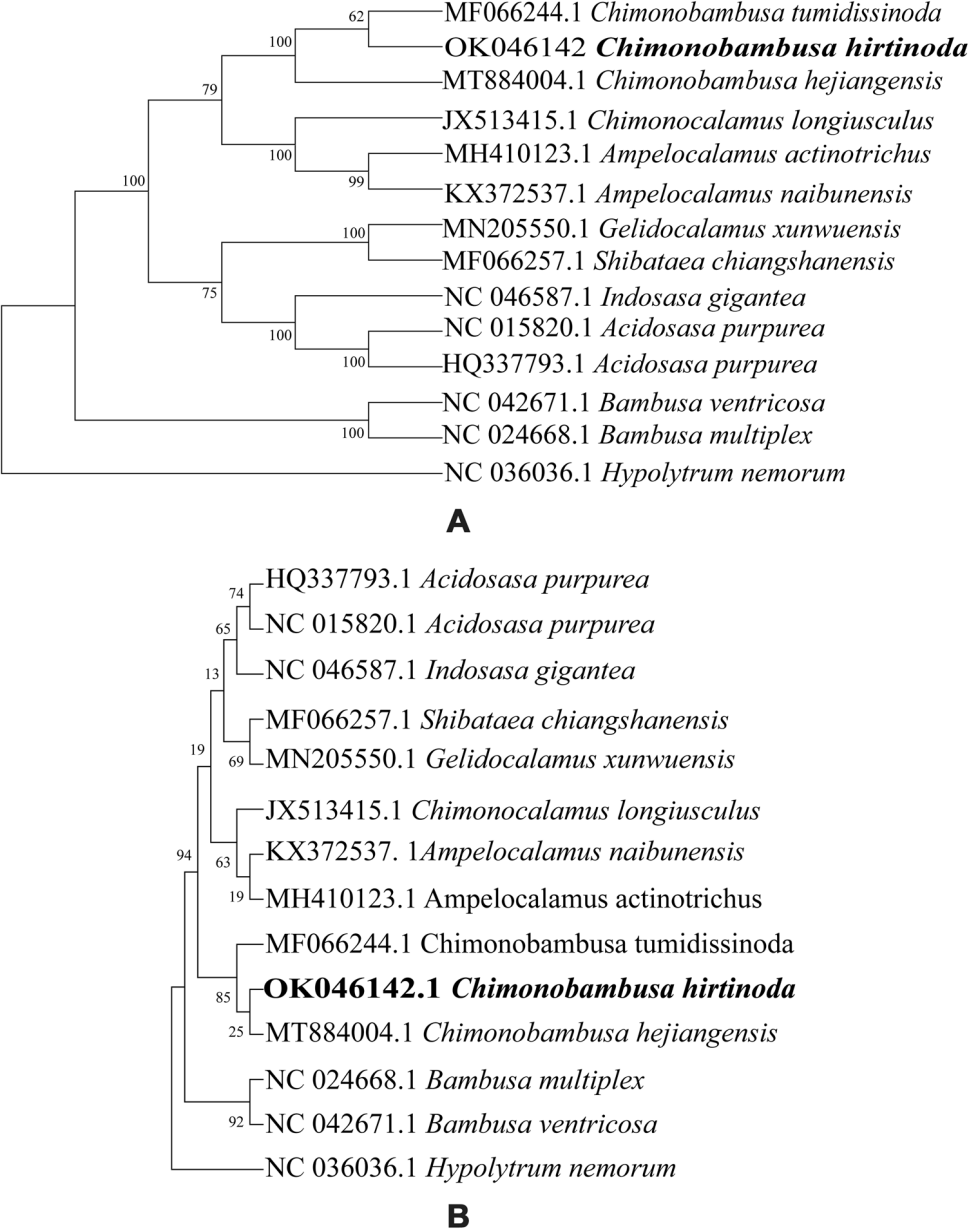
Maximum likelihood phylogenetic tree based on the complete chloroplast genomes (**A**) and *matK* gene (**B**).

We performed a phylogenetic analysis using the complete chloroplast genomes and *matK* gene reflecting the phylogenetic position of *C. hirtinoda*. The maximum likelihood (ML) analysis based on the complete chloroplast genomes indicated seven nodes with entirely branch support (100% bootstrap value). However, the three *Chimonobambusa* genera exhibited a moderate relationship due to fewer samples used, supporting that *C. hirtinoda* is closely related to *C. tumidissinoda* with a 62% bootstrap value more than *C. hejiangensis.* A phylogenetic tree based on the *matK* gene revealed that *Chimonobambusa* species clustered in one branch was consistent with the phylogenetic tree constructed by the complete cp genome tree (Fig. 9). The results show that the whole chloroplast genome identified related species better than the former, consistent with the previous study^39^.

## Discussion

In the current study, *Chimonobambusa* genus exhibited a typical circular tetrad structure, similar to most species. The flowering cycle in bamboo is long and unfixed because of its specific characteristics, and the morphological reproductive and nutritional traits are often difficult to identify because of environmental changes. Therefore, their classification based on morphological details is controversial, leading to unreliable systematic research results^40^. Molecular biology and sequencing technology are more practical methods for their classification. The analysis of plant genetic diversity, species formation, and genetic differentiation is also studied^41^. Therefore, mutation sites observed in the whole chloroplast genome can be used as super bar codes to study the phylogeny and molecular taxonomy of bamboo species.

Genetic diversity is one of the crucial indicators to measure the degree of variation. The higher the degree of genetic diversity, the higher the genetic diversity and abundant genetic resources in the population. Genetic diversity is helpful to improve the adaptability of the species to climate change and historical events and provide scientific and effective strategies for the protection and management of germplasm resources of endangered species. Huang et al. used 16 SSR primers to explore the population genetic diversity and genetic differentiation of the endangered plant *Camellia chekiangoleosa*, and the species conservation strategy was formulated based on the research results^42^.

## Conclusions

The current study primarily explored the chloroplast genome of *C. hirtinoda* and compared it with related species within *Chimonobambusa* genus. These data provide valuable genetic information that advances the genetic research on *Chimonobambusa*. A phenomenon of genes loss was discovered by successfully assembling, annotating, and analyzing the whole chloroplast genome sequence of *C. hirtinoda.* The loss is associated with the rapid evolution of the Poaceae species and the extensive rearrangements of chloroplast structures during the evolutionary process. The acquisition of these data, particularly in terms of SSRs, will enhance the study of the phylogenetic relationships of *Chimonobambusa* plants, their cp genome variation, and gene function.

IR region is the most conserved region of the chloroplast genome. The expansion and contraction of the IR region impact understanding of the evolution of plant population development. The IR region showed no significant difference in the three *Chimonobambusa* species in our current research. In addition, a comparative analysis of the *Chimonobambusa* species revealed that coding regions of the cp genome are more conservative than non-coding regions. Such a change in genetic structure can reflect a relationship with the changes in species, but the mechanism that generates such variations and the subsequent results require further study.

The Poaceae family is generally divided into two large evolutionary branches (BEP and PACCMAD), among which the Bambusoideae, Pooideae, and Oryzoideae belong to the BEP branch. Panicoideae, Arundinoideae, Chloridoideae, Aristidoideae, Arundinoideae, and Micrairoideae belong to the PACCMAD branch. Here, the complete chloroplast genomes based phylogenetic tree (Fig. 9A) revealed high bootstrap support values (only three values less than 80%), and these species can be polymerized into two clades and an outgroup. The genus *Bambusa* constitutes an isolated evolutionary branch, becoming a monophyletic group, a conclusion that is consistent with the previous reports^43^. The two phylogenetic trees revealed that *C. hirtinoda*, *C. hejiangensis* and *C. tumidissinoda* formed a closely related group. In the evolutionary subclade of the second branch, the genus *Ampelocalamus* and *C. longiusculus* have a very close relationship.

## Materials and methods

### Plant materials

**Figure 10:**
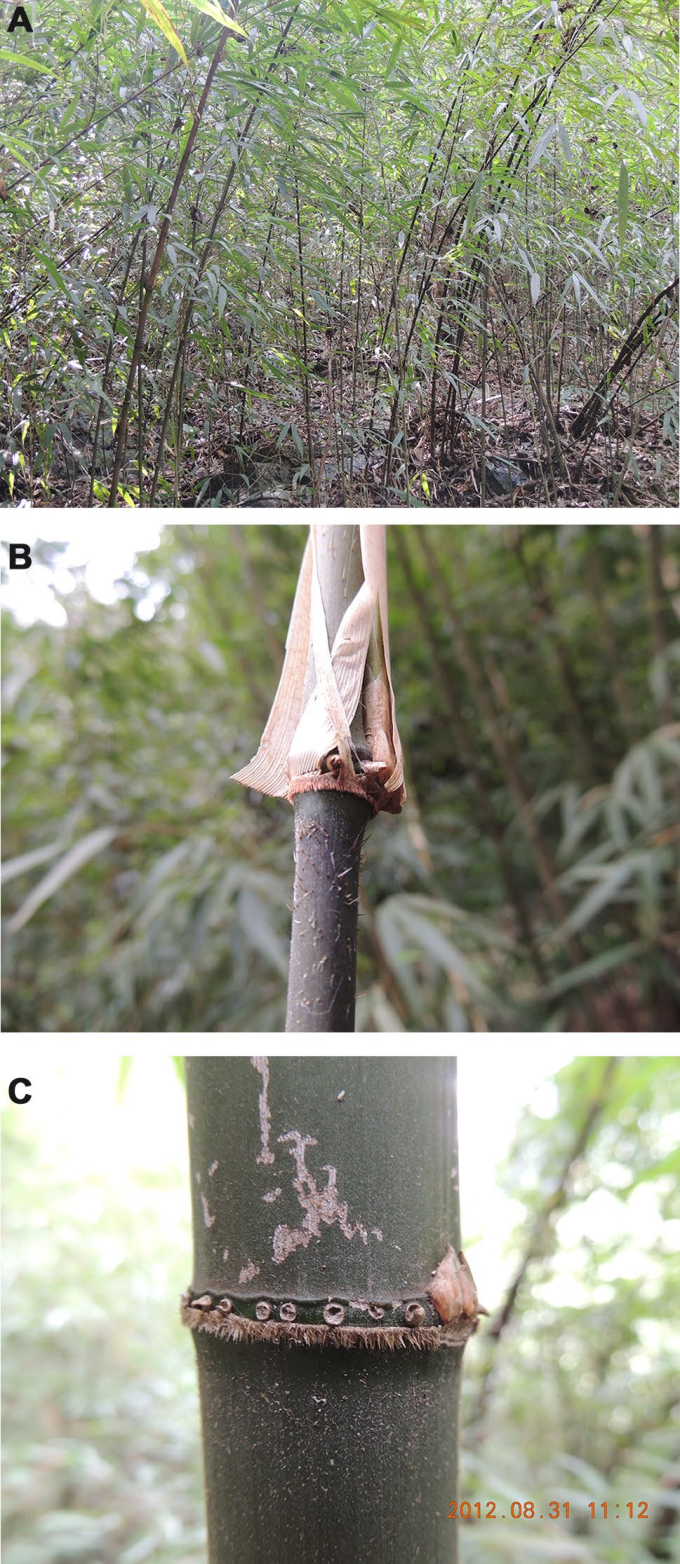
Morphological characteristic of *C. hirtinoda*. (**A**) The wild habitat of *C. hirtinoda*; (**B**) nodal ridges; (**C**) rings of root thorns.

Four to six fresh leaves of *C. hirtinoda* from their native habitats, Doupeng Mountain, Guizhou Province, China (N 26°22′32.55″, E 107°22′9″, altitude 1074.93 m), were collected. The fresh leaves were dried with silica gel and stored at The Natural Museum of Guizhou University (accession number: GACP) for further DNA extraction (Fig. 10). The species identification was performed by Professor Guangqian Gou, the director of the Bamboo Research Institute in the college of life sciences, Guizhou University.

The collection and experiments of plant materials have complied with relevant guidelines and regulations of the Doupeng Mountain virgin forest nature reserve.

### DNA extraction, Chloroplast genome sequencing

Total genomic DNAs were extracted from the sample using the TIANGEN DNA extraction kit (TIANGEN BIOTECH CO., Beijing, China), and the DNA concentration was detected using spectrophotometry. Total DNA quality was detected using 1% agarose gel electrophoresis. All the DNA obtained from *C. hirtinoda* was sent to BGI (Wuhan, China. https://www.genomics.cn), and the total DNA was sequenced using an Illumina sequencer with an HiSeq2500 system, with the library type selected to be the De Novo Sequencing ≤ 800 bp conventional library.

### Genome assembly and annotation

De novo assembly of the *C. hirtinoda* cp genome was performed in a GetOrganelle pipeline^44^ (https://github.com/Kinggerm/GetOrganelle). Using the complete chloroplast genome of *C. hejiangensis* (MT884004) as a reference sequence, PGA (https://github.com/quxiaojian/PGA) annotated the chloroplast genes of *C. hirtinoda,* followed by manual correction using the software Generous 10.0.5^45^. The corrected sequence was uploaded to NCBI. The GenBank accession number of C. hirtinoda was OK046142.

Using the online software package Organellar Genome DRAW (OGDraw)^46^ (http://ogdraw.mpimp-golm.mpg.de/index.shtml), a physical map of the chloroplast group was created.

### Structural of the *C. hirtinoda* cp genome

A simple repeat sequence (SSR) is also called a microsatellite. SSR can be detected using the identification tools MISA^47^ and REPuter^48^. The number for the repeat parameter was set to at least 10, 5, 4, 3, 3, and 3 repeat units, from mononucleotide to hexanucleotide. The codon bias for the chloroplast genome was analyzed using the software package CodonW1.4.2 (http://downloads.fyxm.net/CodonW-76666.html).

### Sequence divergence

Determining the nucleotide diversity of the whole cp genome can make the identification of related species more accurate and help solve similar problems in the phylogenetic research^49,50^. To compare the differences, three species of *Chimonobambusa* were selected, using *C. hejiangensis* as a reference sequence. The software package MAFFT^51^ was used to compare the whole cp genomes of the three species, with the comparison results manually truncated at both ends. Then the software package DnaSP5.10 was used to calculate the Pi values among species sequences^51^. The sliding window was set to 600, and the step size was 200. The online program mVISTA^52^ was used to compare three species, using *C. hejiangensis* annotation as the reference. The software MAUVE^53^ provided rearrangements of those gene sequences.

### Phylogenetic analyses

For 14 sequences of complete chloroplast genome sequences and *matK* gene of Bambusoideae species and *Hypolytrum nemorum* (Cyperaceae: *Hypolytrum*) was selected as an outgroup for the construction of the phylogenetic tree to identify the taxonomic position of the *C. hirtinoda*. All sequences were aligned using the tool MAFFT, and the maximum likelihood (ML) phylogenetic tree was constructed using the software package MEGA-X^54^, and the bootstrap replicates parameter was set to 1000.

### Specimen collection statement

The collection of fresh leaves obtained the permission of the nature reserve.

## Supplementary Information

Supplementary Information 1.

Supplementary Information 2.

Supplementary Information 3.

## Data Availability

The complete chloroplast sequence generated and analyzed during the current study is available in GenBank (https://www.ncbi.nlm.nih.gov, accession numbers are described in the text).
