# Supplementary material for: The complete chloroplast genome of critically endangered Chimonobambusa hirtinoda (Poaceae: Chimonobambusa) and phylogenetic analysis
Source: Sci Rep. 2022 Jun 10;12:9649. doi: 10.1038/s41598-022-13204-2 (PMC9187695; doi:10.1038/s41598-022-13204-2)
Supplement: Supplementary file 1 — Supplementary Information 1. [file 41598_2022_13204_MOESM1_ESM.docx]

| **Gene Functions** | **Gene Family** | **Gene Names** |
| --- | --- | --- |
| Photosynthesis | Subunits of ATP synthase | *atpA, atpB, atpE, atpF*, atpH, atpI* |
|  | Subunits of NADH dehydrogenase | *ndhA*, ndhB*(×2), ndhC, ndhD, ndhE, ndhF, ndhG, ndhH, ndhI, ndhJ, ndhK,* |
|  | Subunits of cytochrome | *petA, petB*, petD*, petG, petL, petN* |
|  | Subunits of photosystem I | *psaA, psaB, psaC, psaI, psaJ* |
|  | Subunits of photosystem II | *psbA, psbB, psbC, psbD, psbE, psbF, psbH, psbI, psbJ, psbK, psbL, psbM, psbN, psbT, psbZ* |
|  | Subunit of rubisco | *rbcL* |
| Other genes | c-type cytochrome synthesis gene | *ccsA* |
|  | Envelop membrane protein | *cemA* |
|  | Protease | *clpP* |
|  | Translational initiation | *infA* |
|  | Maturase | *matK* |
|  | Large subunit of ribosome | *rpl2*(×2), rpl14, rpl16*, rpl20, rpl22, rpl23(×2),* *rpl32, rpl33, rpl36* |
|  | DNA dependent RNA polymerase | *rpoA, rpoB, rpoC1, rpoC2* |
| Self-replication | Small subunit of ribosome | *rps2, rps3, rps4, rps7(×2), rps8, rps11, rps12*(×2), rps14, rps15(×2), rpsl16*, rps18, rps19(×2)* |
|  | rRNA Genes | *rrn23(×2),* *rrn16(×2), rrn5(×2), rrn4.5(×2)* |
|  | tRNA Genes | *trnA-UGC*(×2), trnC-GCA, trnD-GUC, trnE-UUC, trnF-GAA, trnG-GCC, trnG-UCC** , *trnH-GUG(×2), trnI-GAU*(×2) , trnK-UUU, trnL-CAA(×2), trnL-UAA*, trnL-UAG, trnM-CAU, trnN-GUU(×2), trnP-UGG, trnQ-UUG, trnR-ACG(×2), trnR-UCU, trnS-GCU, trnS-GGA,* *trnS-UGA, trnT-GGU, trnT-UGU, trnV-GAC(×2), trnV-UAC*,* *trnW-CCA, trnY-GUA* |
| Unknown function | Conserved open reading frames | *ycf3***, *ycf4*, *ycf68*(×2) |

**Table S1.** List of genes annotated in the cp genomes of *C.* hirtinoda. Genes marked with the sign are the gene with a single (*) or double (**) introns and duplicated genes (×2).
