# Supplementary material for: The complete chloroplast genome of critically endangered Chimonobambusa hirtinoda (Poaceae: Chimonobambusa) and phylogenetic analysis
Source: Sci Rep. 2022 Jun 10;12:9649. doi: 10.1038/s41598-022-13204-2 (PMC9187695; doi:10.1038/s41598-022-13204-2)
Supplement: Supplementary file 2 — Supplementary Information 2. [file 41598_2022_13204_MOESM2_ESM.docx]

| **Number** | **Size** | **Position 1** | **Type** | **Position 2** | **Location1(2)** | **Region** |
| --- | --- | --- | --- | --- | --- | --- |
| 1 | 100 | 14510 | F | 84899 | rpl2 | IRb |
| 2 | 100 | 14510 | P | 137716 | rpl2 | IRa |
| 3 | 63 | 29124 | F | 29325 | rpoC2 | LSC |
| 4 | 62 | 29105 | F | 29306 | rpoC2 | LSC |
| 5 | 62 | 68193 | F | 68214 | rps18 | LSC |
| 6 | 58 | 68207 | F | 68228 | rps18 | LSC |
| 7 | 56 | 29135 | F | 29336 | rpoC2 | LSC |
| 8 | 55 | 29215 | F | 29257 | rpoC2 | LSC |
| 9 | 52 | 104154 | P | 104154 | rps12 | IRb |
| 10 | 52 | 104154 | F | 118509 | rps12 | IRa |
| 11 | 52 | 118509 | P | 118509 | rps12 | IRa |
| 12 | 54 | 16337 | F | 16404 | trnT-GGU | LSC |
| 13 | 54 | 29098 | F | 29374 | rpoC2 | LSC |
| 14 | 50 | 29157 | F | 29199 | rpoC2 | LSC |
| 15 | 44 | 68221 | F | 68242 | rps18 | LSC |
| 16 | 46 | 13566 | F | 38710 | trnfM-CAU | LSC |
| 17 | 42 | 29114 | F | 29390 | rpoC2 | LSC |
| 18 | 32 | 58643 | P | 85489 | rpl23 | IRb |
| 19 | 32 | 58643 | F | 137194 | rpl23 | IRa |
| 20 | 41 | 13474 | F | 38614 | trnfM-CAU | LSC |
| 21 | 41 | 29395 | F | 29473 | rpoC2 | LSC |
| 22 | 38 | 29232 | F | 29274 | rpoC2 | LSC |
| 23 | 40 | 29171 | F | 29213 | rpoC2 | LSC |
| 24 | 37 | 29119 | F | 29473 | rpoC2 | LSC |
| 25 | 39 | 29095 | F | 29296 | rpoC2 | LSC |
| 26 | 30 | 18660 | P | 18660 | psbM | LSC |
| 27 | 30 | 68614 | P | 68614 | rps18 | LSC |
| 28 | 30 | 89823 | P | 89823 | ndhB | IRb |
| 29 | 30 | 89823 | F | 132862 | ndhB | IRa |
| 30 | 30 | 132862 | P | 132862 | ndhB | IRa |
| 31 | 36 | 8392 | P | 47037 | trnS-GGA | LSC |
| 32 | 36 | 13576 | F | 38720 | rps14 | LSC |
| 33 | 36 | 29374 | F | 29440 | rpoC2 | LSC |
| 34 | 36 | 45561 | F | 92828 | ycf3 | LSC |
| 35 | 36 | 45561 | P | 129851 | ycf3 ( rps12) | LSC (IRa) |
| 36 | 38 | 29173 | F | 29257 | rpoC2 | LSC |
| 37 | 38 | 40656 | F | 42880 | psaB (psaA) | LSC |
| 38 | 32 | 13409 | F | 13576 |  | LSC |
| 39 | 32 | 13409 | F | 38720 |  | LSC |
| 40 | 32 | 29093 | F | 29189 | rpoC2 | LSC |
| 41 | 37 | 29320 | F | 29395 | rpoC2 | LSC |
| 42 | 37 | 68207 | F | 68249 | rps18 | LCS |
| 43 | 36 | 29098 | F | 29440 | rpoC2 | LSC |
| 44 | 30 | 13608 | F | 38584 |  | LSC |
| 45 | 35 | 13458 | F | 38598 | trnfM-CAU | LSC |
| 46 | 35 | 15168 | F | 92175 | rps12 | IRb |
| 47 | 35 | 15168 | P | 130505 | rps12 | IRa |
| 48 | 32 | 15374 | P | 22330 | rpoB | LSC |
| 49 | 32 | 29325 | F | 29478 | rpoC2 | LSC |
| 50 | 32 | 58670 | P | 85461 | rpl23 | IRb |
| 51 | 32 | 58670 | F | 137222 | rpl23 | IRa |
| 52 | 34 | 16364 | F | 16431 | trnT-GGU | LSC |
| 53 | 34 | 29199 | F | 29358 | rpoC2 | LSC |
| 54 | 31 | 29180 | F | 29222 | rpoC2 | LSC |
| 55 | 30 | 29098 | F | 29152 | rpoC2 | LSC |
| 56 | 32 | 12043 | P | 47044 | trnS-UGA (trnS-GGA) | LSC |
| 57 | 32 | 16367 | P | 48265 | trnT-GGU | LSC |
| 58 | 31 | 29117 | F | 29426 | rpoC2 | LSC |
| 59 | 31 | 29393 | F | 29426 | rpoC2 | LSC |
| 60 | 31 | 82371 | F | 82416 | rps3 | LSC |
| 61 | 30 | 104181 | F | 118536 | ndhD | IRa |

**Table S2** Distribution and localization of repetitive sequences in *C. hirtinoda* chloroplast genome.
