# Supplementary material for: The complete chloroplast genome of critically endangered Chimonobambusa hirtinoda (Poaceae: Chimonobambusa) and phylogenetic analysis
Source: Sci Rep. 2022 Jun 10;12:9649. doi: 10.1038/s41598-022-13204-2 (PMC9187695; doi:10.1038/s41598-022-13204-2)
Supplement: Supplementary file 3 — Supplementary Information 3. [file 41598_2022_13204_MOESM3_ESM.docx]

| **AA** | **Codon** | **No.** | **RSCU** | **tRNA** | **AA** | **Codon** | **No.** | **RSCU** | **tRNA** |
| --- | --- | --- | --- | --- | --- | --- | --- | --- | --- |
| Phe | UUU | 721 | 1.29 |  | Ser | UCU | 384 | 1.58 |  |
|  | UUC | 395 | 0.71 | *trnF-GAA* |  | UCC | 294 | 1.21 | *trnS-GGA* |
| Leu | UUA | 698 | 1.93 | *trnL-UAA* |  | UCA | 249 | 1.02 | *trnS-UGA* |
|  | UUG | 401 | 1.11 | *trnL-CAA* |  | UCG | 133 | 0.55 |  |
|  | CUU | 459 | 1.27 |  | Pro | CCU | 325 | 1.49 |  |
|  | CUC | 163 | 0.45 |  |  | CCC | 215 | 0.98 | *trnP-GGG* |
|  | CUA | 330 | 0.91 | *trnL-UAG* |  | CCA | 239 | 1.09 |  |
|  | CUG | 119 | 0.33 |  |  | CCG | 96 | 0.44 |  |
| Ile | AUU | 816 | 1.49 |  | Thr | ACU | 454 | 1.69 |  |
|  | AUC | 322 | 0.59 | *trnI-GAU* |  | ACC | 194 | 0.72 | *trnT-GGU* |
|  | AUA | 501 | 0.92 | *trnI-CAU* |  | ACA | 299 | 1.11 | *trnT-UGU* |
| Met | AUG | 478 | 1.00 | *TrnfM-CAU* |  | ACG | 129 | 0.48 |  |
| Val | GUU | 431 | 1.45 |  | Ala | GCU | 544 | 1.73 |  |
|  | GUC | 146 | 0.49 | *trnV-GAC* |  | GCC | 190 | 0.60 |  |
|  | GUA | 446 | 1.50 | *trnV-UAC* |  | GCA | 371 | 1.18 | *trnA-UGC* |
|  | GUG | 166 | 0.56 |  |  | GCG | 152 | 0.48 |  |
| Tyr | UAU | 572 | 1.58 |  | Cys | UGU | 171 | 1.55 |  |
|  | UAC | 154 | 0.42 | *trnY-GUA* |  | UGC | 49 | 0.45 | *trnC-GCA* |
| TER | UAA | 45 | 1.59 |  | TER | UGA | 21 | 0.74 |  |
|  | UAG | 19 | 0.67 |  | Trp | UGG | 347 | 1.00 | *trnW-CCA* |
| His | CAU | 342 | 1.47 |  | Arg | CGU | 288 | 1.36 | *trnR-ACG* |
|  | CAC | 122 | 0.53 | *trnH-GUG* |  | CGC | 109 | 0.52 |  |
| Gln | CAA | 519 | 1.54 | *trnQ-UUG* |  | CGA | 266 | 1.26 |  |
|  | CAG | 155 | 0.46 |  |  | CGG | 96 | 0.45 |  |
| Asn | AAU | 587 | 1.48 |  | Ser | AGU | 294 | 1.21 |  |
|  | AAC | 205 | 0.52 | *trnN-GUU* |  | AGC | 107 | 0.44 | *trnS-GCU* |
| Lys | AAA | 739 | 1.43 | *trnK-UUU* | Arg | AGA | 372 | 1.76 | *trnR-UCU* |
|  | AAG | 294 | 0.57 |  |  | AGG | 136 | 0.64 |  |
| Asp | GAU | 558 | 1.54 |  | Gly | GGU | 480 | 1.26 |  |
|  | GAC | 166 | 0.46 | *trnD-GUC* |  | GGC | 153 | 0.40 |  |
| Glu | GAA | 777 | 1.47 | *trnE-UUC* |  | GGA | 600 | 1.57 | *trnG-UCC* |
|  | GAG | 281 | 0.53 |  |  | GGG | 293 | 0.77 |  |

**Table S3.** Comparative Analysis of chloroplast codon usage bias of *C.* *hirtinoda*
